# Psychosocial and Financial Impact of Dermatophytosis on Patients Attending a Tertiary Care Centre at Visakhapatnam: A Cross-Sectional Study

**DOI:** 10.7759/cureus.82483

**Published:** 2025-04-18

**Authors:** Priyanka Karanam, Venkata Dileep Kumar Veldi, Arun Kumar Metta, Sri Sai Praneeth Angara, Neeraja Rani, Pandrakula Sandhya, Sarath Chandra Ponnada, Dhwani A Mehta

**Affiliations:** 1 Medicine, Gayatri Vidya Parishad Institute of Health Care and Medical Technology, Visakhapatnam, IND; 2 Dermatology, Gayatri Vidya Parishad Institute of Health Care and Medical Technology, Visakhapatnam, IND; 3 Pharmacology, Great Eastern Medical School and Hospital, Srikakulam, IND; 4 Community Medicine, Great Eastern Medical School and Hospital, Srikakulam, IND; 5 General Medicine, Great Eastern Medical School and Hospital, Srikakulam, IND

**Keywords:** dermatology life quality index, dermatophytosis, financial burden, psychosocial impact, quality of life (qol)

## Abstract

Background

Dermatophytosis has become a growing concern in India, with rising cases fueled by recurrent infections, antifungal resistance, and misuse of steroid-antifungal treatments. Beyond the physical discomfort, many patients struggle with embarrassment, depression, and a diminished quality of life (QoL). This study bridges that gap by using the Dermatology Life Quality Index (DLQI) questionnaire to assess how dermatophytosis affects patients' daily lives, while also exploring the financial strain it imposes, offering valuable insights to improve care and address the challenges these individuals face.

Methodology

This cross-sectional study was conducted over four months in the department of Dermatology, Venereology, and Leprosy at Gayatri Vidya Parishad Institute of Healthcare and Medical Technology, Visakhapatnam, involving 123 patients aged ≥18 years diagnosed with dermatophytosis. Patients with tinea who provided informed consent were included following our inclusion and exclusion criteria. Data collection utilized the DLQI questionnaire and case study forms to capture sociodemographics, clinical variables, and financial burden. Data were analyzed using SPSS version 25 with descriptive statistics and Chi-square tests. Ethical approval and written consent were obtained.

Results

The study, conducted among 123 participants aged 18 to 74 years (mean age 39.07 ± 12.72), highlighted the substantial burden of dermatophytosis on the QoL. Tinea corporis, alone or with tinea cruris, was the most common diagnosis (41; 33.3%), and most cases occurred in the 30-45 age group. The majority of patients, 104 (85%), experienced a prolonged disease duration of six to 24 months. Despite varying lesion counts and sites, including the pubic region, face, and extremities, dermatophytosis consistently showed a significant psychosocial impact, with 74 (60%) reporting a "very large" and 35 (28%) an "extremely large" effect on the DLQI. Gender differences were negligible, as both males and females experienced comparable impacts. Financial strain and worry were common among participants, with 32 (26%) being financially dependent, and higher worry levels correlating with worse DLQI scores. Although statistical significance was not achieved for some variables, the findings underline the pervasive and multifaceted challenges faced by dermatophytosis patients.

Conclusion

This study underscores the significant psychosocial and financial burden of dermatophytosis on affected individuals, particularly those in the most productive age group of 30-45 years. Prolonged disease duration and widespread lesion distribution highlight the chronic and pervasive nature of the condition. The DLQI revealed that the majority of patients experienced a "very large" or "extremely large" impact on their QoL, irrespective of gender, number of lesions, or diagnosis type. Although financial dependency and worry levels were notable, they showed a close yet statistically insignificant association with the QoL impact. These findings highlight the importance of timely diagnosis and appropriate treatment to reduce the prolonged suffering associated with dermatophytosis. Addressing both the physical symptoms and the emotional and financial challenges faced by patients is crucial for improving their overall QoL. A patient-centered approach, focusing on comprehensive care and support, can significantly alleviate the burden of this condition.

## Introduction

Dermatophytosis has emerged as a critical public health issue in India, with cases escalating to near-epidemic proportions in recent years, with an estimated 20-25% of the world's population. Many individuals endure recurrent infections that are resistant to conventional treatments, often exacerbated by the misuse of steroid creams, poor hygiene practices, and inappropriate antifungal medication use [1,2]. Despite the availability of expert guidelines, such as those issued by the Expert Consensus on the Management of Dermatophytosis in India (ECTODERM India), which outline protocols for handling resistant cases, the challenges in patient care extend beyond pharmacological solutions [3]. The effect of dermatophytosis on patients' quality of life (QoL) is frequently overlooked in clinical settings, despite the condition causing notable emotional and social hardship.

Coping with dermatophytosis presents significant challenges, as individuals often grapple with physical discomfort alongside emotional repercussions like embarrassment, depression, and, in severe cases, suicidal ideation due to the chronic and resistant nature of these infections [1,4]. While QoL assessment tools such as the Dermatology Life Quality Index (DLQI) have been extensively utilized for skin conditions like psoriasis and vitiligo, their application in dermatophytosis remains limited, resulting in a significant gap in understanding the lived experiences and unmet needs of affected individuals [5,6]. Factors such as environmental conditions, the presence of strains like *Trichophyton mentagrophyte*, and heightened inflammatory responses further contribute to the persistence and recurrence of infections [7]. The misuse of combination steroid treatments and the increasing resistance to antifungal drugs complicate management efforts even further [2,8].

Recognizing the inadequacy of current research on the QoL implications, dermatology organizations across India have emphasized the need for focused investigation into this area [9]. In addition, the financial burden of managing recurrent, resistant dermatophytosis poses a significant challenge for patients, encompassing repeated doctor visits, prolonged medication regimens, and indirect costs such as productivity loss. Considering the profound social and emotional toll of dermatophytosis, evaluating quality of life is vital for a holistic approach to treatment. Addressing these psychological and social dimensions could lead to better outcomes, surpassing the boundaries of physical health alone. This study aimed to fill this gap by analyzing the impact of dermatophytosis on patients' quality of life using the DLQI questionnaire and assessing the financial strain it imposes.

Aims and objectives

This study aimed to evaluate the impact of dermatophytosis on patients' QoL using the DLQI and to assess the financial burden, including both direct and indirect costs, among affected individuals.

## Materials and methods

Study design, setting, and duration

A hospital-based observational cross-sectional study was conducted among patients diagnosed with dermatophytosis to assess the QoL in the Department of Dermatology, Venereology and Leprosy (DVL) at Gayatri Vidya Parishad Institute of Health Care and Medical Technology (GVPIHCMT), a tertiary healthcare center in Madhurawada, Visakhapatnam, over a period of four months to assess the QoL among patients diagnosed with dermatophytosis.

Sample size

A total of 123 participants were selected using purposive sampling based on average outpatient attendance during the study period and meeting the inclusion criteria. A total of 140 patients were diagnosed with tinea, but 123 participants were included in the study based on the predefined inclusion and exclusion criteria.

Study population and selection criteria

The study included all patients aged 18 years and above diagnosed with dermatophytosis attending a tertiary healthcare center in Visakhapatnam. Both males and females diagnosed with tinea who provided informed consent were included. We included both naïve and previously treated patients, regardless of treatment stage. Patients below 18 years, those with other skin disorders, individuals on medication for other medical or surgical conditions, pregnant and lactating women, and illiterate patients were excluded from the study.

Data collection

The study utilized a structured DLQI questionnaire to assess the QoL. A case study form was used to collect data on sociodemographic characteristics and clinical variables such as duration, site and number of lesions, and the financial dependency, burden, and worry experienced by patients. The DLQI questionnaire and case study form with financial aspects are mentioned in the appendix (see Appendices A and B). Financial burden and worry scales adapted from the original work by Veenstra et al. [10]. Data collection was done by the DLQI questionnaire, translated into the local language (Telugu) and administered (Appendix C).

Data analysis

Data were entered into Microsoft Excel (Microsoft Corp., WA, USA) and analyzed using IBM SPSS Statistics for Windows, Version 25.0 (released 2017, IBM Corp., Armonk, NY). Descriptive statistics such as mean, standard deviation, frequency, and percentage were used to summarize sociodemographic and clinical characteristics. The associations between variables such as sex, diagnosis, and number of lesions with DLQI scores were assessed using Chi-square tests, with a p-value of <0.05 considered statistically significant.

Institutional Ethics Committee (IEC) and consent

The study was conducted following approval from the Institutional Ethical Committee (IEC) of GVPIHCMT, Marikavalasa, Visakhapatnam, under reference number RC. No: GVPIHCMT/IEC/20231003/05, with written informed consent obtained from all participants.

## Results

The study included 123 participants, whose ages ranged from 18 to 74 years, with a mean age of 39.07 years (SD = 12.72). Although age was not found to be a significant factor influencing the occurrence of dermatophytosis, the majority of cases were observed in the 30-45 age group, indicating that this demographic was the most affected.

Among 123 participants, 70 (56.9%) were male, and 53 (43.1%) were female, indicating a slight male predominance. DLQI scores showed that 62 (88.5%) males and 45 (85%) females experienced a very large or extremely large psychosocial impact from dermatophytosis. However, sex was not significantly associated with the severity of impact (p = 0.4) (Table 1).

**Table 1:** Distribution of study participants by sex, dermatophytosis diagnosis, and number of lesions DLQI: Dermatology Life Quality Index Pearson's chi-squared test; p < 0.001: highly significant; p < 0.05: significant

Variable	Category	DLQI interpretation			Chi-square value	p-value
		Moderate (n, %)	Very large (n, %)	Extremely large (n, %)		
Sex	Male	8 (11.4%)	40 (57.1%)	22 (31.4%)	1.83	0.4
	Female	8 (15.1%)	34 (64.2%)	11 (20.8%)		
Diagnosis	Tinea corporis	2 (7.1%)	18 (64.3%)	8 (28.6%)	7	0.86
	Tinea mannum	1 (14.3%)	4 (57.1%)	2 (28.6%)		
	Tinea cruris	4 (15.4%)	16 (61.5%)	6 (23.1%)		
	Tinea barbae	0 (0%)	2 (50.0%)	2 (50.0%)		
	Tinea facei	0 (0%)	9 (81.8%)	2 (18.2%)		
	Tinea corporis + Tinea cruris	8 (19.5%)	22 (53.7%)	11 (26.8%)		
	Tinea pedis	1 (16.7%)	3 (50.0%)	2 (33.3%)		
No. of lesions	1	1 (7.7%)	6 (46.2%)	6 (46.2%)	7.76	0.8
	2	3 (8.6%)	21 (60.0%)	11 (31.4%)		
	3	7 (18.4%)	24 (63.2%)	7 (18.4%)		
	4	4 (17.4%)	13 (56.5%)	6 (26.1%)		
	5	1 (9.1%)	7 (63.6%)	3 (27.3%)		
	6	0 (0%)	2 (100.0%)	0 (0%)		
	7	0 (0%)	1 (100.0%)	0 (0%)		

**Figure 1:**
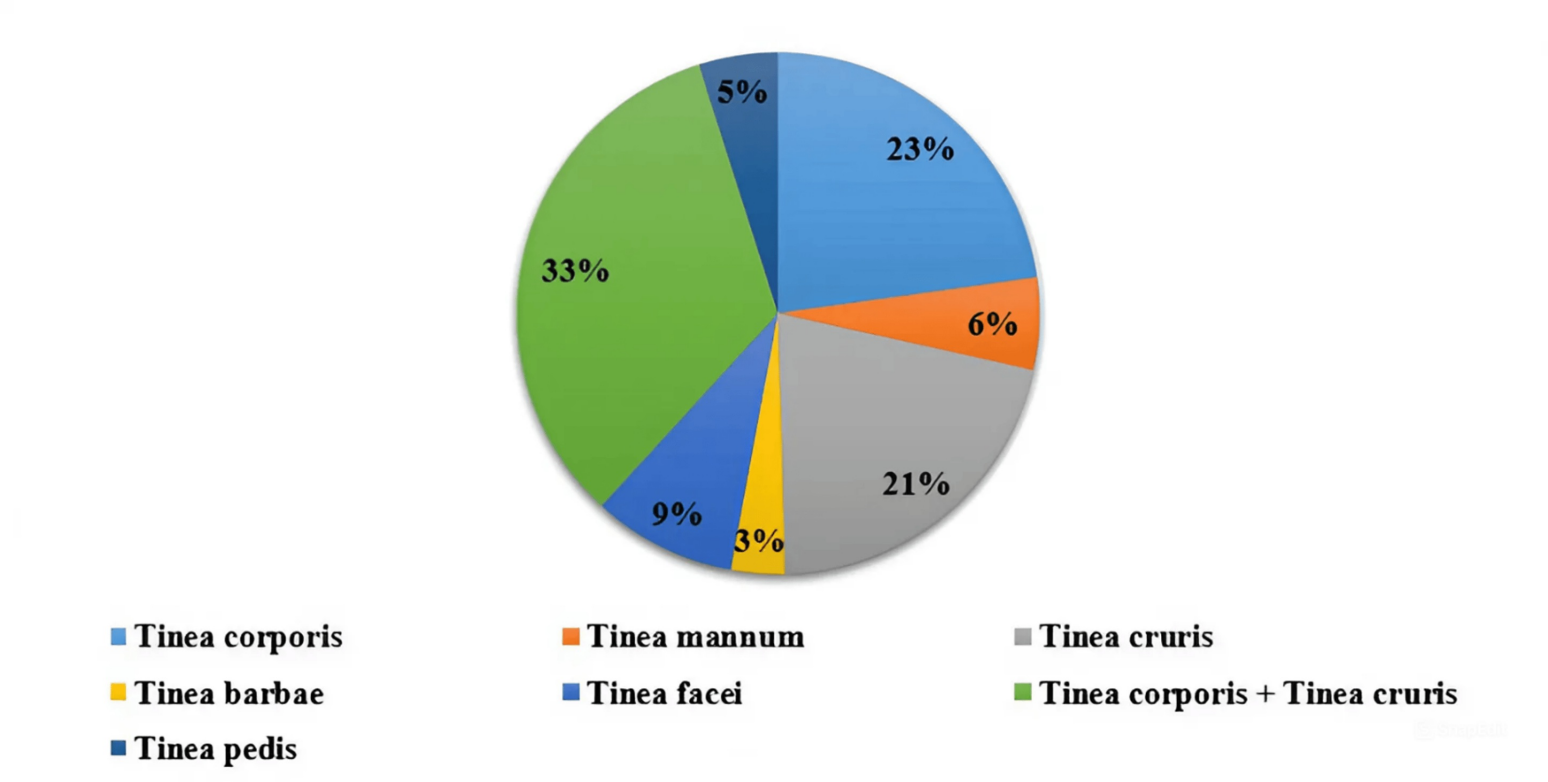
Tinea diagnosis distribution

In our study, tinea corporis (with or without tinea cruris) emerged as the most common diagnosis, accounting for 41 (33.3%) cases, followed by tinea cruris at 26 (21.1%), highlighting the prevalence of trunk and groin infections. Less common diagnoses included tinea manuum (seven; 5.7%) and tinea pedis (six; 4.9%). Although various diagnoses had a notable impact on the QoL as indicated by DLQI scores, with many participants reporting a "very large" impact, there was no statistically significant association between diagnosis type and QoL impact (p = 0.86) (Figure 1).

In our study, the majority presented with multiple lesions, with 35 (28.5%) reporting two lesions and 38 (30.9%) reporting three, while only 13 (10.6%) had a single lesion, reflecting a range in disease severity. Interestingly, the analysis showed no statistically significant association between lesion count and DLQI scores (p = 0.8), suggesting that the number of lesions may not directly correlate with psychosocial impact in dermatophytosis.

Most patients (104; 85%) had dermatophytosis for six to 24 months, with 50 (41%) having it for six to 12 months and 54 (44%) for 12 to 24 months. Only 16 (13%) had it for less than six months and two (2%) for over 24 months. This indicates a prolonged duration of dermatophytosis in most patients (Table 2).

**Table 2:** Duration of onset for dermatophytosis cases

Duration	Frequency	Percent
0-6 months	16	13%
6-12 months	50	41%
12-24 months	54	44%
>24 months	3	2%
Total	123	100%

In our study, lesion sites varied widely, with the pubic region being the most frequently affected, followed by the face. Other commonly affected areas included the hands, abdomen, chest, legs, and back, highlighting the diverse presentations of tinea across different body regions (Table 3).

**Table 3:** Distribution of lesions in dermatophytosis

Site of lesion	Frequency	Percent
Left hand	4	3%
Palm.	7	6%
Front abdomen+ pubic region	2	2%
Front abdomen	2	2%
Front chest+ pubic region	8	7%
Right hand+ pubic region	2	2%
Pubic region	22	18%
Back abdomen + pubic region	9	7%
Buttocks + pubic region	3	2%
Left leg	3	2%
Breast + pubic region	2	2%
Beard + moustache	4	3%
Plantar aspect of the feet	6	5%
Breast	11	9%
Face	4	3%
Buttock	4	3%
Right hand + pubic region	5	4%
Neck + pubic region	3	2%
Left leg + pubic region	4	3%
Back abdomen	4	3%
Neck	5	4%
Front chest	5	4%
Back chest + pubic region	4	3%
Total	123	100%

**Figure 2:**
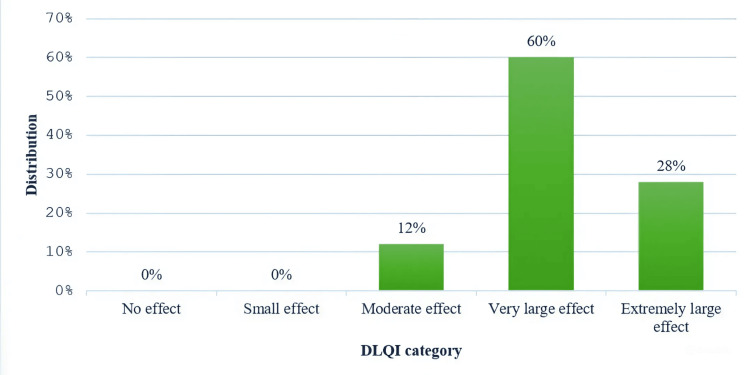
DLQI interpretation DLQI: Dermatology Life Quality Index

DLQI scores among the participants ranged from 10 to 29, with a mean score of 17.45 (SD = 4.00), reflecting the varying impact of dermatophytosis on the QoL. A majority (74; 60%) reported a very large effect, while 35 (28%) experienced an extremely large impact, underscoring the significant psychosocial burden associated with the condition. Notably, none of the participants reported a negligible or small effect (Figure 2).

Financial burden

The mean financial burden in our study was 3.43 (SD = 0.93), while the mean financial worry score was 3.34 (SD = 1.01). Among 123 total participants, 32 patients (26%) were financially dependent, while the remaining 92 patients (74%) were financially independent.

The financial burden associated with dermatophytosis and its impact on the QoL (DLQI). A significant proportion of patients reported a "very large" impact across most categories, particularly in category 2 with 81 (66%) and category 4 with 82 (67%). While financial burden varied across categories, the association between financial burden and DLQI was close to significant (p = 0.06) (Table 4).

**Table 4:** Association between financial burden and financial worry with DLQI interpretation DLQI: Dermatology Life Quality Index Pearson's chi-squared test; p < 0.001: highly significant; p < 0.05: significant

Variable	Category	DLQI interpretation			Chi-square value	p-value
		Moderate (6–10)	Very large (11–20)	Extremely large (21–30)		
Financial burden	1	1 (100%)	0 (0%)	0 (0%)	17.7	0.06
	2	2 (8%)	16 (66%)	6 (25%)		
	3	7 (24%)	13 (44%)	9 (31%)		
	4	6 (10%)	40 (67%)	13 (22%)		
	5	0 (0%)	5 (55%)	4 (44%)		
	6	0 (0%)	0 (0%)	1 (100%)		
	7	0 (0%)	0 (0%)	0 (0%)		
Worry	1	1 (50.0%)	1 (50.0%)	0 (0%)	10.61	0.22
	2	3 (11.5%)	16 (61.5%)	7 (26.9%)		
	3	5 (12.5%)	21 (52.5%)	14 (35.0%)		
	4	2 (5.4%)	27 (73.0%)	8 (21.6%)		
	5	5 (27.8%)	9 (50.0%)	4 (22.2%)		

The distribution of worry levels due to dermatophytosis and their corresponding impact on the QoL (DLQI). Most individuals reporting higher worry levels (scales 3 and 4) experienced a "very large" impact on DLQI, with 64 (52.5%) and 90 (73.0%), respectively. However, the association between worry levels and DLQI impact remains statistically insignificant (p = 0.22).

## Discussion

In our study of 123 participants aged 18 to 74 years (mean age 39.07, SD = 12.72), the majority of cases were observed in the 30-45 age group, suggesting this demographic was most affected. However, age did not significantly influence the occurrence of dermatophytosis. In contrast, Patel et al. [11] found the highest prevalence in a younger demographic, with the majority of respondents in the 18-30 age range. This difference in age distribution might reflect variations in exposure or health-seeking behaviors across age groups.

In our study, the majority of participants experienced a significant impact on their quality of life due to dermatophytosis, with 74 (60%) reporting a "very large" effect and 34 (28% reporting an "extremely large" effect on the DLQI, resulting in a mean DLQI score of 17.45 (SD = 4.00). This suggests a substantial psychosocial burden among patients at our center. In comparison, Patel et al. [11] reported a lower mean DLQI score of 12.25 (SD = 5.56) among 299 respondents, with 153 (51.5%) experiencing a very large impact and 29 (9.7%) an extremely large impact. Similarly, Mushtaq et al. [9] observed a mean DLQI of 13.4 (SD = 7.3) in a cohort of 348 patients, with 155 (44.8%) reporting a very large effect. Verma et al. [12], however, noted a lower mean DLQI score of 8.2 (SD = 5.1) and found that only 26.3% experienced very large or extremely large effects. The differences in DLQI scores across studies could be attributed to variations in sample demographics, clinical severity, or regional factors affecting psychosocial responses to dermatophytosis.

Among the 123 participants in our study, 70 (56.9%) were male and 53 (43.1%) were female, showing a slight male predominance. DLQI scores indicated that 62 (88.5%) of males and 60 (85%) of females experienced a very large or extremely large psychosocial impact from dermatophytosis, but no significant association was found between sex and the severity of impact (p = 0.4). Patel et al. [11] reported similar findings, with a marginal male majority (151 males vs. 148 females) and no significant difference in DLQI scores between sexes. Likewise, Patro et al. [13] observed nearly equal numbers of male and female patients, supporting the notion that dermatophytosis impacts the QoL similarly across genders. However, Verma et al. [12] found that females had slightly higher DLQI scores than males (9.3 ± 5.2 vs. 7.1 ± 4.7, p = 0.038), suggesting that women may experience a somewhat greater psychosocial impact in certain populations. Differences in psychosocial perceptions or cultural factors could contribute to this variance in impact across studies.

In our study, tinea corporis (with or without tinea cruris) was the most common diagnosis, observed in 23 (33.3%) cases, followed by tinea cruris alone at 15 (21.1%), highlighting the frequent involvement of the trunk and groin regions. Less common diagnoses included tinea manuum (40; 5.7%) and tinea pedis (34; 4.9%). While various diagnoses had a notable impact on the QoL, with many participants reporting a very large impact on their DLQI scores, there was no statistically significant association between diagnosis type and QoL impact (p = 0.86). Similarly, Patel et al. [11] identified tinea corporis with tinea cruris as the leading presentation, affecting 65.55% of their study group, with tinea corporis alone in 16.05% and tinea cruris alone in 8.03% of cases. Patro et al. [11] also observed high frequencies of tinea corporis (237 cases) and tinea cruris (225 cases). Verma et al. [12] noted that more than half of their patients presented with tinea corporis and tinea cruris combined, with fewer instances of standalone presentations. Although our study found no significant link between specific diagnoses and QoL impact, trends suggest that tinea corporis and tinea cruris, whether alone or combined, may have a pronounced effect on QoL.

In our study, the majority of participants presented with multiple lesions, with 35 (28.5%) reporting two lesions and 38 (30.9%) reporting three, while only 13 (10.6%) had a single lesion. This range in lesion count reflects variability in disease severity. However, there was no statistically significant association between the number of lesions and DLQI scores (p = 0.8), suggesting that lesion count may not directly correlate with the psychosocial impact of dermatophytosis. By contrast, Mushtaq et al. [9] reported a significant effect of lesion count on DLQI scores (p < 0.001), and Das et al. [14] found that having more than one site involved was a risk factor for higher DLQI scores.

In our study, lesion sites varied widely, with the pubic region being the most frequently affected, followed by the face. Other commonly affected areas included the hands, abdomen, chest, legs, and back, highlighting the diverse presentations of tinea across different body regions.

In comparing our findings to those of Patel et al. [11], where 80.36% of patients reported using savings for treatment, we observed that financial burden significantly impacted QoL, with 81 (66%) of patients in category 2 and 82 (67%) in category 4 experiencing a "very large" impact on the DLQI. This highlights a comparable trend of substantial financial strain, although Patel et al. focused on direct cost considerations. Similarly, Jamil et al. [15] reported mean financial burden and worry scores of 3.79 ± 1.82 and 3.29 ± 1.10, respectively, and found a weak, non-significant correlation with DLQI scores. In our study, while the association between financial burden and DLQI was close to significance (p = 0.06), the statistically insignificant association between worry and DLQI (p = 0.22) aligns with Jamil et al.'s [15] findings, suggesting variability in how financial strain and worry directly influence the QoL. This comparison emphasizes the multifaceted nature of financial and emotional stress in dermatophytosis patients.

Limitations

This study has several limitations. The cross-sectional design precludes causal inferences, and the single-center setting may limit generalizability to broader populations. In addition, financial burden assessment relied on self-reported data, which may be subject to recall bias. The lack of standardized tools for assessing financial strain further limits comparability across studies. The statistical correlation between different age groups may change with a higher number of the study population, considering wider demographic area with more people from all age groups. Future research should employ longitudinal designs and larger, more diverse populations to validate these findings.

Generalizability

Despite its limitations, this study provides valuable insights into the psychosocial and financial impacts of dermatophytosis. The findings are likely applicable to tertiary care settings in similar demographic and geographic regions but may not generalize to community-based populations or other healthcare systems.

## Conclusions

This study highlights that dermatophytosis is far more than a superficial skin condition; it significantly disrupts the lives of those affected. The chronic and recurrent nature of the disease imposes a substantial emotional and financial strain on patients, particularly within the 30-45 age group, where the QoL is most severely impacted. Many individuals expressed feelings of embarrassment, frustration, and even hopelessness, as persistent and treatment-resistant infections interfered with their daily routines, personal relationships, and social activities. Conditions such as tinea corporis and tinea cruris, especially when involving visible or sensitive areas, were found to carry a profound emotional burden. Patients consistently reported feelings of isolation and distress, regardless of differences in the size or location of lesions. 

Compounding this challenge, the financial burden of repeated treatments is often exacerbated by improper steroid use, and growing antifungal resistance adds another layer of difficulty, not only affecting the patients but also placing a strain on their families. The findings emphasize that successful management of dermatophytosis must go beyond addressing physical symptoms. It is equally essential to consider the emotional and social challenges faced by patients. By fostering a holistic approach that includes education about appropriate treatment methods and support for mental health, healthcare providers can help patients regain control of their lives and alleviate the often-overlooked suffering associated with the condition. Future research should aim to explore the long-term psychosocial and economic effects of dermatophytosis across diverse populations and develop strategies to enhance treatment accessibility and outcomes. A multidisciplinary approach is crucial to addressing not only the physical manifestations of the disease but also the broader emotional and social impacts, ultimately leading to improved patient care.

## Figures and Tables

**Figure 3:**
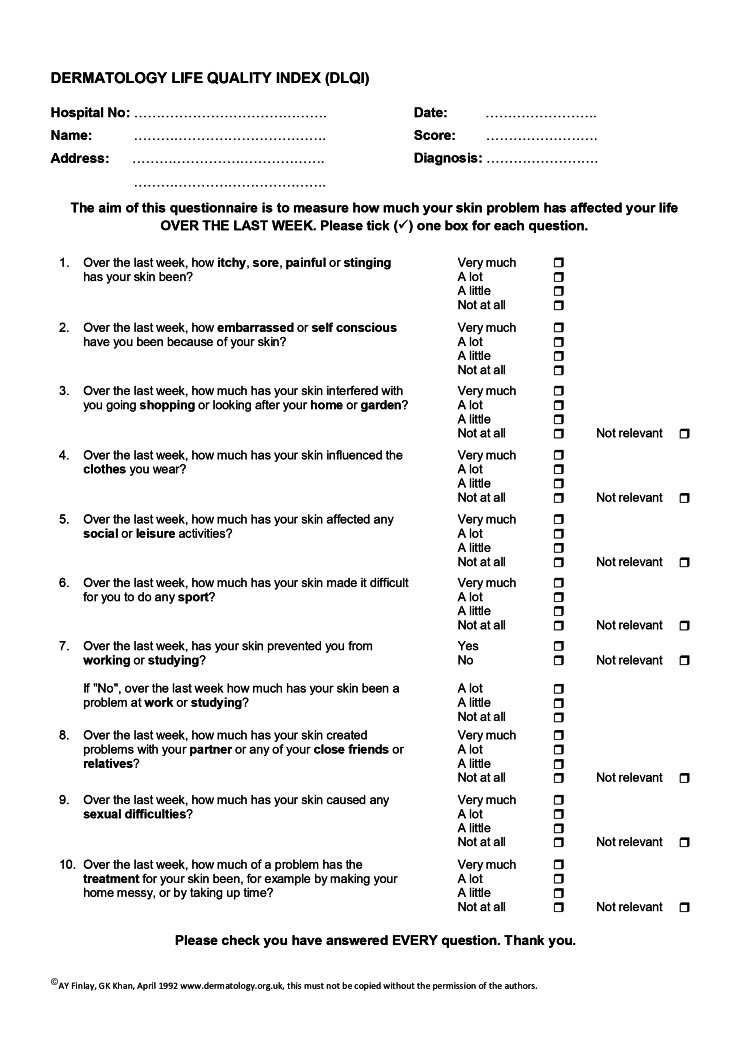
DLQI questionnaire Image Source: Finlay and Khan, 1994 [4]. We have obtained permission from the original publisher "Oxford University Press and Copyright Clearance Center" DLQI: Dermatology Life Quality Index

**Figure 4:**
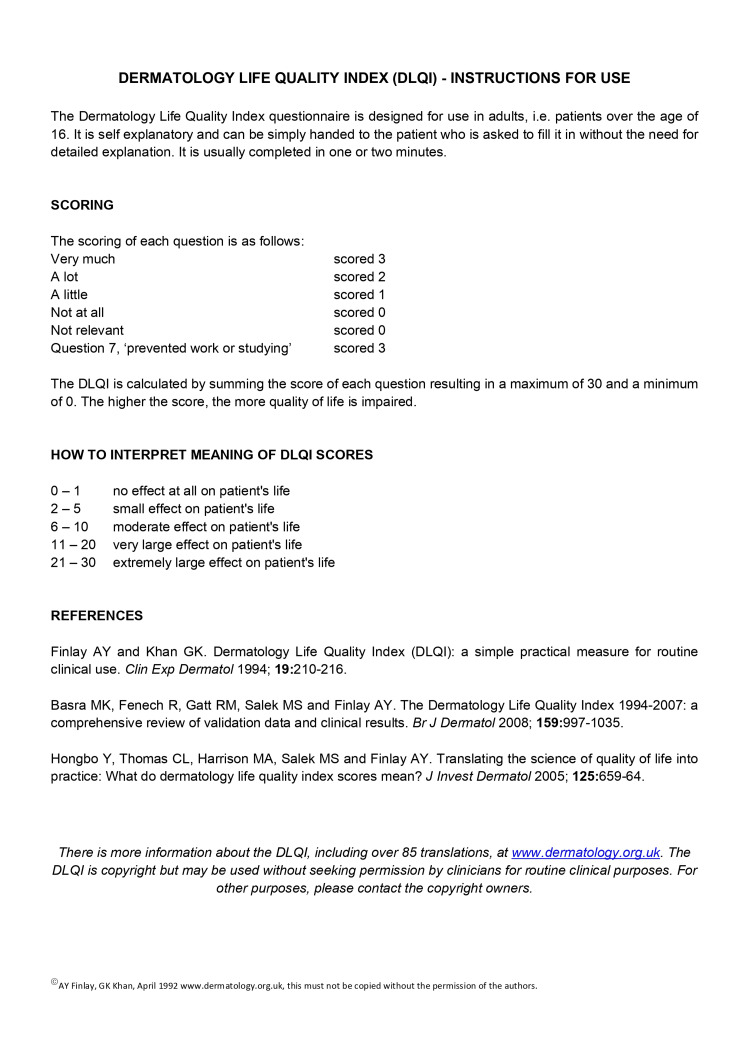
DLQI questionnaire Image Source: Finlay and Khan, 1994 [4]. We have obtained permission from the original publisher "Oxford University Press and Copyright Clearance Center" DLQI: Dermatology Life Quality Index

**Figure 5:**
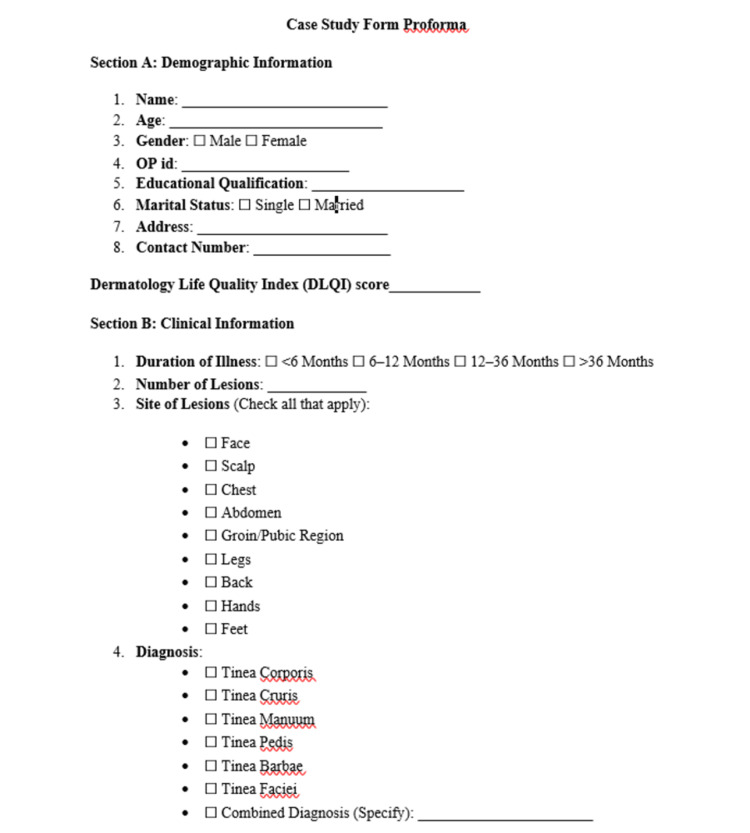
Case study proforma

**Figure 6:**
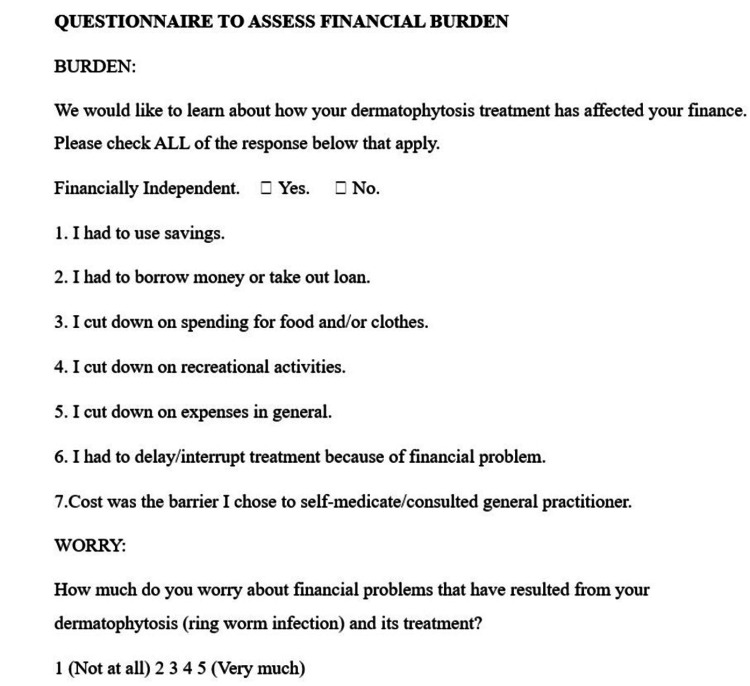
Scale for financial burden and worry

## Appendices

Appendix A

Appendix B

Appendix C

## References

[1] Panda S, Verma S (2017). The menace of dermatophytosis in India: the evidence that we need. Indian J Dermatol Venereol Leprol.

[2] Verma S, Madhu R (2017). The great Indian epidemic of superficial dermatophytosis: an appraisal. Indian J Dermatol.

[3] Rajagopalan M, Inamadar A, Mittal A (2018). Expert consensus on the management of dermatophytosis in India (ECTODERM India). BMC Dermatol.

[4] Finlay AY, Khan GK (1994). Dermatology Life Quality Index (DLQI)--a simple practical measure for routine clinical use. Clin Exp Dermatol.

[5] Weitzman I, Summerbell RC (1995). The dermatophytes. Clin Microbiol Rev.

[6] Sahoo AK, Mahajan R (2016). Management of tinea corporis, tinea cruris, and tinea pedis: a comprehensive review. Indian Dermatol Online J.

[7] Dogra S, Uprety S (2016). The menace of chronic and recurrent dermatophytosis in India: is the problem deeper than we perceive?. Indian Dermatol Online J.

[8] Narang T, Mahajan R, Dogra S (2017). Dermatophytosis: fighting the challenge: conference proceedings and learning points. September 2-3, 2017, PGIMER, Chandigarh, India. Indian Dermatol Online J.

[9] Mushtaq S, Faizi N, Amin SS, Adil M, Mohtashim M (2020). Impact on quality of life in patients with dermatophytosis. Australas J Dermatol.

[10] Veenstra CM, Regenbogen SE, Hawley ST (2014). A composite measure of personal financial burden among patients with stage III colorectal cancer. Med Care.

[11] Patel NH, Padhiyar JK, Patel AP, Chhebber AS, Patel BR, Patel TD (2020). Psychosocial and financial impact of disease among patients of dermatophytosis, a questionnaire-based observational study. Indian Dermatol Online J.

[12] Verma S, Vasani R, Reszke R, Matusiak L, Szepietowski JC (2021). The influence of superficial dermatophytoses epidemic in India on patients' quality of life. Postepy Dermatol Alergol.

[13] Patro N, Panda M, Jena AK (2019). The menace of superficial dermatophytosis on the quality of life of patients attending referral hospital in Eastern India: a cross-sectional observational study. Indian Dermatol Online J.

[14] Das A, Sil A, Fatima F, Podder I, Jafferany M (2022). Impact of chronic and recurrent dermatophytosis on quality of life and psychologic morbidity-a cross-sectional study. J Cosmet Dermatol.

[15] Jamil R, Saxena K, Koti V, Mohanty S (2023). Dermatophytosis: its impact on quality of life and financial burden. Int J Med Biomed Stud.

